# Une cause rare de détresse respiratoire du nourrisson: l'angiome sous glottique

**DOI:** 10.11604/pamj.2014.17.43.3564

**Published:** 2014-01-22

**Authors:** Smael Labib, Mustapha Harandou

**Affiliations:** 1 Service de Réanimation Mère et Enfant, CHU Hassan II, Fès, Maroc

**Keywords:** Angiome sous-glottique, nourrisson, tumeur bénigne, laryngite, Subglottic hemangioma, infant, benign tumor, laryngitis

## Image en medicine

L'angiome sous-glottique du nourrisson appartient au groupe des hémangiomes immatures du nourrisson. C'est une tumeur bénigne qui résulte d'une prolifération cellulaire clonale de l'endothélium capillaire. Il doit systématiquement être évoqué chez le nourrisson de moins de six mois devant un tableau de laryngite sous-glottique, sensible à la corticothérapie, mais pour laquelle des signes laryngés persistent après traitement ou récidivent dans les jours qui suivent. L'imagerie est pau contributive au diagnostic. L'endoscopie met en évidence l'hémangiome: une tuméfaction molle, sous une muqueuse d'aspect plus ou moins angiomateux ou congestif. Le traitement était basé sur la corticothérapie et la chirurgie, il fait maintenant essentiellement appel au propranolol, qui permet de limiter considérablement le nombre de gestes chirurgicaux. Nous rapportons le cas d'un nourrisson de 8 semaines sans antécédents particuliers, qui a présenté de façon progressive une dyspnée à prédominance inspiratoire, un cornage aux pleurs et un tirage intercostal, il a reçu une corticothérapie et une antibiothérapie à base d'amoxicilline et acide clavulanique. La symptomatologie s'est aggravée progressivement pendant une semaine ayant entrainée une détresse respiratoire, avec un balancement thoraco-abdominal et une cyanose. Le nourrisson a bénéficié alors d'une trachéotomie en urgence. Le bilan étiologique a montré à la fibroscopie bronchique: un bombement de la paroi postérieure de la trachée à 5 mm du plan glottique en faveur d'un angiome sous glottique. Le patient a été mis sous propanolol pendant 3 mois, la fibroscopie de contrôle a montré une régression presque complète de l'angiome.

**Figure 1:**
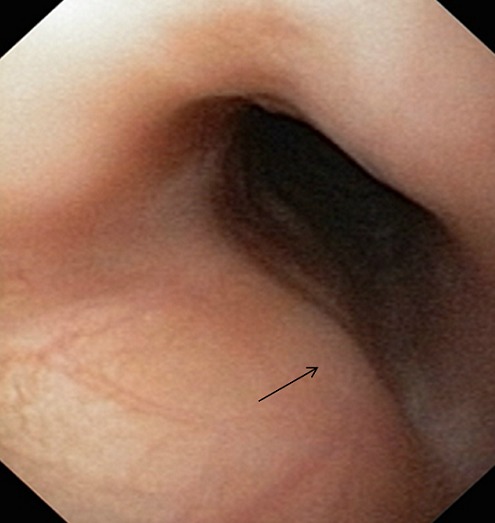
Bombement de la paroi postérieure de la trachée à 5 mm du plan glottique en faveur d'un angiome sous glottique

